# Exploring How Sociocultural Factors Affect the Experience of Completing Neuropsychological Assessments Within Older Greek-Australians

**DOI:** 10.1093/arclin/acad044

**Published:** 2023-06-17

**Authors:** Joyce Rizkallah, Mathew Staios, Penelope Analytis, Mary H Kosmidis, Evrim March, Renerus J Stolwyk

**Affiliations:** Turner Institute for Brain and Mental Health, School of Psychological Sciences, Monash University, Melbourne, Australia; Monash-Epworth Rehabilitation Research Centre, Monash University, Melbourne, Australia; Turner Institute for Brain and Mental Health, School of Psychological Sciences, Monash University, Melbourne, Australia; Monash-Epworth Rehabilitation Research Centre, Monash University, Melbourne, Australia; Turner Institute for Brain and Mental Health, School of Psychological Sciences, Monash University, Melbourne, Australia; Monash-Epworth Rehabilitation Research Centre, Monash University, Melbourne, Australia; Lab of Cognitive Neuroscience, School of Psychology, Aristotle University of Thessaloniki, Thessaloniki, Greece; Turner Institute for Brain and Mental Health, School of Psychological Sciences, Monash University, Melbourne, Australia; Turner Institute for Brain and Mental Health, School of Psychological Sciences, Monash University, Melbourne, Australia; Monash-Epworth Rehabilitation Research Centre, Monash University, Melbourne, Australia

**Keywords:** Greek Australian, Cultural neuropsychology, Neuropsychological assessment

## Abstract

**Objective:**

The field of cultural neuropsychology has grown exponentially over the last three decades. With a limited culturally informed evidence base to guide neuropsychological practice, the acceptability of existing paradigms has been called into question when applied to culturally diverse and educationally disadvantaged groups. This qualitative study aimed to explore the experiences of Greek Australian older adults who underwent a cognitive assessment to better understand potential barriers and facilitators to engagement and to improve neuropsychological assessment outcomes.

**Method:**

Semi-structured interviews were developed to explore cultural attitudes and contextual factors relating to neuropsychological assessment. Interviews were conducted by Greek-speaking neuropsychologists using a sample of 10 healthy elderly Greek Australians following the completion of a comprehensive neuropsychological assessment. Data were analyzed using a phenomenological design within a critical realist framework.

**Results:**

Analysis revealed the emergence of three broad themes: sociocultural factors, experiences within the broader medical system, and the assessment experience. Engagement with cognitive assessment was influenced by several factors, including rapport building, understanding of the assessment, and use of inappropriate tests. Furthermore, level and quality of education, sex differences, language barriers, acculturation, previous experiences of prejudice, anxiety, and a preference for Greek-speaking clinicians were additional factors reported to affect the client experience and validity of assessment outcomes.

**Conclusion:**

Neuropsychological assessment is, in part, affected by culturally reinforced attitudes. Failing to adjust the relationship between the clinician and client, test environment, style of communication, and the use of culturally inappropriate tests is likely to affect the validity of assessment outcomes.

## Introduction

As of 2020, there were over 55 million people worldwide living with dementia. This number is expected to almost double every 20 years, reaching approximately 139 million by 2050 (World Health Organisation, 2022). Projected trends suggest that over 700,000 Australian older adults are expected to meet the criteria for dementia by 2050, with one-third consisting of individuals from culturally and linguistically diverse backgrounds (Australian Institute of Health & Welfare, 2018). Greek Australian immigrants represent one of the largest aging culturally and linguistically diverse groups in Australia, totaling approximately 63,000 individuals aged 65 years and over, with over 40,000 having achieved a primary level of education or less (Australian Bureau of Statistics, 2016; Fanany & Avgoulas, 2019). Neuropsychological assessment plays a central role in the diagnosis and management of neurocognitive disorders, including dementia (Pimentel, 2009). The clinical utility of neuropsychological assessment is, to a large extent, dependent on the acceptability and validity of neuropsychological tests (Strauss, Sherman, & Spreen, 2006). Research has established that neuropsychological tests and norms developed for majority populations are prone to misclassification when applied to culturally diverse minority groups (Carstairs, Myors, Shores, & Fogarty, 2006; Daugherty, Puente, Fasfous, Hidalgo-Ruzzante, & Pérez-Garcia, 2017; Nielsen & Staios, 2023). In contrast, further research originating from South Africa has indicated that mixed-group norms can lead to misclassifying educationally disadvantaged culturally and linguistically diverse majority groups as impaired due to poor test specificity and inadequate representation in normative samples (Pienaar, Shuttleworth-Edwards, Klopper, & Radloff, 2016; Shuttleworth-Edwards, 2016; Shuttleworth-Edwards et al., 2004). Factors such as cultural and linguistic heterogeneity, level and quality of education, use of unrepresentative normative data, unfamiliar test content, and test-taking attitudes have been noted to affect testing outcomes (Rivera Mindt, Byrd, Saez, & Manly, 2010; Shuttleworth-Edwards, 2016; Staios, 2022).

In addition to the aforementioned factors, research has also proposed that cultural attitudes and contextual factors may exert a significant impact on test-taking outcomes (Ardila, 2007). For example, Ardila (2005) and Fujii (2018) argue that rapport building, language and communication style, the testing environment, and orientation toward psychometric testing have the potential to affect the validity of test outcomes. Relative to majority groups, individuals from culturally and linguistically diverse minority groups have been reported to bring a different set of expectations and values to the assessment process, which has been attributed to differences in educational styles and socially reinforced attitudes (Rosselli & Ardila, 2003). Research conducted with healthy educationally disadvantaged Greek Australian immigrants noted that factors such as level of education, test-taking skills, and level of acculturation resulted in poorer performances on cognitive screening instruments (Plitas et al. 2009). In a related study, Veliu and Leathem (2016) observed a range of issues when assessing culturally diverse populations in New Zealand, including those from Asia, Africa, and the Middle East. Despite following a range of available recommended guidelines, they reported encountering multiple barriers, including difficulties establishing rapport and maintaining communication between client and clinician, using interpreters with little prior experience in neuropsychological assessment, culturally inappropriate tests, and clients not comprehending formal test instructions (Veliu & Leathem, 2016). Similar issues were observed by Aghvinian and colleagues (2020) in a sample of Xhosa-speaking South African adults, who had no prior experience with cognitive assessment. Although most participants found testing procedures acceptable, many were not able to grasp the concept of neuropsychological testing, some provided culture-bound explanations as to why cognitive impairment may occur (e.g., witchcraft), and none were familiar with the term dementia. Furthermore, participants identified several other issues that affected their assessment experience, including mistaking standard procedures as indicative of poor performance (e.g., no feedback from the examiner and stopped due to time limits), the use of inappropriate tests (e.g., drawing tests, abstract reasoning, and arithmetic), and their level and quality of education (Aghvinian et al., 2020).

Although past research has provided both observational theories and preliminary qualitative findings concerning factors that may additionally affect the validity of neuropsychological test outcomes, surprisingly limited empirical research has examined this issue. Therefore, further research across multiple international contexts is required to ascertain the potential contribution of cultural attitudes and contextual factors from the perspective of culturally diverse individuals undergoing neuropsychological assessment. To address the previously mentioned limitations, this qualitative study aimed to explore the experiences of Greek Australian older adults undergoing neuropsychological assessment for the first time; examine the cultural attitudes and contextual factors that may affect the validity of assessment outcomes; and provide practical strategies to assist with conducting a culturally informed neuropsychological assessment.

## Methods

### Participants

Participants were recruited from a broader study standardizing neuropsychological tests within an older Greek Australian cohort. Inclusion and exclusion criteria are described in detail elsewhere (Staios, Kosmidis, Kokkinis, et al., 2023, Staios, Kosmidis, Nielsen, et al., 2023). In brief, a total of 90 participants completed a neuropsychological test battery that was administered in the Greek language by bilingual Greek–English speaking clinical neuropsychologists. Testing occurred over two sessions, lasting approximately 2 hours in total. Participants were aged between 70 and 85 years, literate, and Greek was their dominant language.

The present study was conceptualized approximately mid-way through data collection for the broader study described previously. Participants who had recently completed neuropsychological assessment were invited to participate. Purposive sampling was used to examine across information-rich cases related to Greek Australian older individuals undergoing neuropsychological assessment for the first time. To achieve a broad and balanced understanding of cultural attitudes within this specific population, we considered age, sex, and years in Australia.

### Procedure

The study was approved by the Monash University Human Research Ethics Committee. The research team developed an open-ended semi-structured interview schedule, based on their clinical and research experiences, while also referring to the cross-cultural neuropsychological literature (Ardila, 2005, 2007). The interview schedule focused on participants’ experience of neuropsychological assessment and included questions regarding test content, administration processes, and the test environment. Refer to the Appendix for the interview schedule.

Participants were interviewed in Greek by bilingual Greek–English-speaking clinical neuropsychologists, authors MS (four interviews) and PA (six interviews). Administration of cognitive assessment measures in the normative study was conducted by a different neuropsychologist in a separate earlier session. Nine interviews were conducted in the participants’ homes and one by telephone. Interviews were audio recorded and lasted between 29 and 60 min (*M* = 38.49, *SD* = 9.65). All interviews took place between July 2019 and September 2019.

### Data Analysis

Audio recordings of interviews were transcribed and translated from Greek into English by a bilingual Greek-English-speaking neuropsychologist who was not part of the research team (YT). Following transcription, MS reviewed all transcripts for accuracy. MS and PA reviewed translation queries to identify differences in Greek–English meaning, semantic equivalence, and grammatical errors, as well as to achieve the most accurate culturally and linguistically equivalent translation. All transcripts were deidentified and subsequently analyzed using NVivo Version 12 (QSR International, 2018), a qualitative data analysis package.

The study utilized a phenomenological design within a critical realist framework, acknowledging that participants’ experiences were understood as their lived reality, situated within social, cultural, and language contexts but simultaneously having a basis in reality (Braun & Clarke, 2021). Data analysis was guided by Braun and Clarke’s (2021) six-phase reflexive thematic approach. First, JR familiarized herself with the data, read the transcripts and field notes numerous times, consulted with authors MS and PA, and compiled familiarization notes allowing the assumptions that underpin analytic observations to be explored. Second, JR used NVivo to code all transcripts according to semantic themes, relating to the explicit and surface meaning of the raw data. Interviews were then coded according to their implicit meanings to capture underlying ideas, patterns, or assumptions. Codes were discussed and revised multiple times by the research team to create a codebook. Third, the research team collaboratively searched for candidate themes to make sense of the data through several meetings, which included input from neuropsychologists (RS and MS) and JR. Fourth, candidate themes were reviewed and refined at an individual and entire sample level, where JR reviewed each transcript and updated final codes to ensure that the codes were consistently applied. Fifth, themes were defined and grouped according to their content by the research team, leading to the final phase of preparing the report of presenting the findings.

To enhance analytic rigor, the authors reflected on criteria for good qualitative research (Braun & Clarke, 2021; Tracy, 2010; Yardley, 2016). Transcripts were checked, linguistic nuances evaluated, coding was thorough, and themes were reviewed against each other and the data. The researchers were actively situated in data collection, analysis, and interpretation. In addition, the author team engaged in critical reflection of their own identities and biases relating to the collection and analysis of the data. They were cognizant of their cultural and professional biases and preexisting beliefs that may have influenced their interpretation of the data. They attempted to mitigate the impact of these biases and beliefs on the analyses by continually referring to the data and the research questions.

## Results

### Participants

Although a total of 16 participants were initially invited to take part in the present study, four participants withdrew due to scheduling conflicts and two withdrew due to caregiving duties. The final sample consisted of 10 healthy Greek Australian older adults. Based on the specificity of the study aim, the homogeneity of participant characteristics and the richness of interviews, the final sample size was considered to provide sufficient information power (Morse, 2015).

Ten Greek Australian older adults (seven women and three men) agreed to participate in the present study. Demographic data for all 10 participants are presented in Table 1 using pseudonyms. In summary, age ranged from 70 to 82 years (*M* = 75.30, *SD* = 4.05), education ranged from 6 to 12 years, with only one participant completing high school (*M* = 6.60, *SD* = 1.89). The average time in Australia was 54 years (*M* = 54.50, *SD* = 2.67). The average time elapsed between completing neuropsychological testing and the qualitative interview was approximately 3 months (*M* = 85.90, *SD* = 39.78; Range = 44–137 days).

**Table 1:** Participant demographic characteristics (*N* = 10)

Pseudonym	Age	Sex	Education (years)	Years in Australia
Kristos	73	M	6	55
Ariadne	71	F	6	51
Alexandra	74	F	6	56
Mary	74	F	6	55
Iris	70	F	6	51
Zoe	79	F	6	57
George	75	M	12	51
Helen	81	F	6	54
Peter	82	M	6	58
Katerina	74	F	6	57

*Note:* F = female; M = male.

### Thematic Analysis

**Fig. 1:**
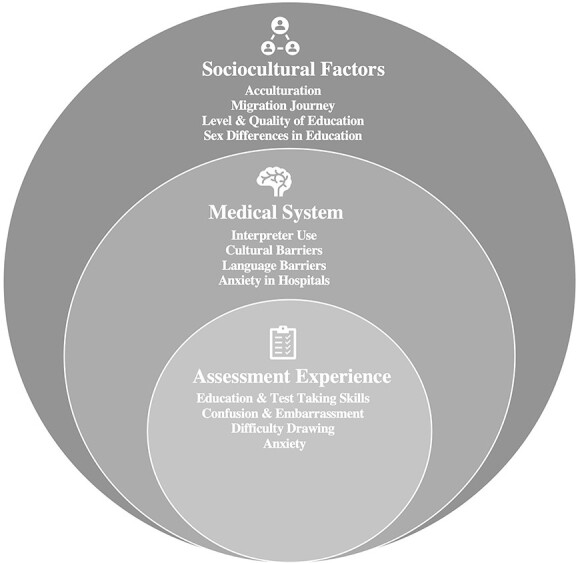
Concentric circles representing the three main themes and their corresponding subthemes.

Three broad themes and several corresponding subthemes were identified relating to the experience of Greek Australian older adults undergoing neuropsychological assessment. These included sociocultural context: acculturation, migration journey, level and quality of education, sex differences, and education; broader medical experiences: interpreter use, cultural barriers, language barriers, and anxiety in hospitals; and experience of neuropsychological assessment: anxiety, confusion, embarrassment, education, and test-taking skills. The broad themes, along with their corresponding subthemes, are presented in Fig. 1.

In summary, the experience of neuropsychological assessment and experiences within the broader medical setting is shaped by sociocultural factors. Overall, participants described some aspects of the assessment as “fun” and “game-like,” however, they characterized other aspects of the process as anxiety provoking and confusing. They were also challenged by their beliefs regarding the effect of their limited level of education and difficulty completing specific tasks, leaving them disheartened. Although efforts were made to inform participants regarding the reason for the assessment, some were not able to grasp the purpose of undergoing cognitive assessment, equating the overall process to a brain game or simple memory test. The themes are further discussed in the following sections, including illustrative quotes from participant interviews.

### Sociocultural Factors

Participants disclosed a range of sociocultural factors, ranging from sex, early education, their migration journey, subsequent cultural belonging, and how these factors shaped the social setting within which the assessment took place. Many participants shared how their educational history may have affected the assessment process. Almost all participants (*n* = 9) reported having achieved a limited formal education and poor quality of schooling, relative to their Australian-born counterparts. Participants were conscious of, and seemed discouraged by, their limited knowledge and how this factor affected the testing experience: “What can I tell you? I am uneducated. I only attended primary school. I am not clever. We were born only to work, and for nothing else (sighing)” (Zoe). One participant reported that the impact of two consecutive wars affected her ability to regularly attend school: “I didn’t attend school for very long. In Greece, at that time, we had war. And then...the war ended, but I had grown too old. I was 16 years old…I was taken to work in the fields, to dig…I hardly learned to write and read” (Helen). Several participants reported that their limited formal education and no previous experience with test-taking negatively affected the assessment process: “I have never done tests like this before. It must be like that in schools in Australia. We did not do this in Greece when I was young” (Kristos).

Participants reported that such unfamiliarity affected their confidence during testing, particularly when they compared themselves to others: “Some of my friends have gone to high school, and some of them read books, they are smarter and educated. People that haven’t finished primary school might be less confident in testing. These tests take confidence” (Iris). Indeed, six participants commented on the impact of reading and self-education, stating that they believed those who were well-read or received further education would have an advantage during neuropsychological assessments. However, all participants felt that such opportunities were scarce for many Greek immigrants: “Greek immigrants that are at an old age and came here 50 or 60 years ago… We were focused only on work and poverty” (Alexandra). In contrast, the only participant who had graduated from high school reflected that he had been exposed to similar test procedures and was confident in his responses: “The tasks were easy because I finished secondary school” (George). George further discussed how the content that was assessed was not taught in primary school, and those that had not had the opportunity to continue schooling would be disadvantaged if required to undergo cognitive assessment.

Many participants expected differences in test-taking skills between men and women. The majority (*n* = 6) reported that men were either smarter or would outperform women. This was largely attributed to sociocultural norms, where education for women was reported to interfere with household duties and traditional gender roles: “A man will do better [on cognitive testing] because they went to school more. Women did not. They stayed at home and looked after the family and cooked” (Katerina). Sex was also seen to have an impact on how comfortable people would be with different clinicians. One female participant said that the sex of the administrator may influence the assessment experience, stating that perhaps a female clinician for a female client would facilitate the assessment, as women may feel shy toward men.

Participants also divulged parts of their challenging migration journey to Australia and the subsequent fortification of their cultural identity. Most participants (*n* = 9) considered themselves Greek first and foremost, preferred to speak Greek, and socialized solely within the Greek community. Reflecting on their arrival to Australia, many experienced prejudices: “I remember when I first came to this country. I couldn’t speak English and when I spoke in Greek, [Australians] would call us stupid wogs. It was a hard time. We kept to ourselves and didn’t want to socialize with Australians because we felt like outsiders. I still feel this way a little” (Mary).

Participants reported that their challenging migration journey led to fortifying trust within their community. This is magnified by participants valuing the preservation of their Greek cultural heritage, and as such were reluctant to integrate into mainstream Australian culture: “We try never to change. We go somewhere, but we don’t forget our country” (Peter). All but one participant reported a preference to be around other Greek Australians. Most participants reported greater levels of trust, comfort, and sense of safety among other Greeks. “I see my husband when we go to [the] hospital, the second a Greek person speaks …He feels more comfortable and your eyes open… You have more trust in someone that’s speaking to you in Greek” (Iris).

The impact of these experiences was also reflected in several ways concerning their experiences of cognitive assessment. On the one hand, participants expressed a sense of safety and trust in response to the presence of a Greek neuropsychologist: “If you hear Greek, your mind takes you back to Greece and you feel safe… We came here very young and all we had was each other, so we relied on each other” (Katerina). On the other hand, participants reported that the assessment content may have been unfamiliar because of their lack of familiarity and experience with Australian culture. Indeed, one participant who felt he was well integrated into Australian culture reported that this aided in the cognitive assessment: “Personally, I am Greek, but I am more into English culture. I read English newspapers. I knew everything he [the neuropsychologist] asked me” (George).

### Broader Medical Setting

Participants shared a range of experiences relating to the broader medical system, such as language barriers and cultural differences, which influenced their expectations and concerns during the cognitive assessments. Half of the participants reported prior negative hospital experiences and difficulties finding clinicians with whom they were comfortable. These experiences were often related to difficulties communicating and a perceived lack of value being placed on their Greek cultural identity: “In hospitals, they do not care that much … and they don’t appreciate us” (Zoe). Five participants found hospitals to be anxiety provoking, either due to fears about being hospitalized, a loss of autonomy, or being misunderstood by healthcare professionals: “Going into hospital makes me feel anxious. It also makes my husband feel anxious” (Iris). Participants shared a range of factors that affected their experiences with clinicians, including limited knowledge of Greek culture and a manner suggesting a lack of warmth and patience. Participants found it difficult to feel at ease when the clinician’s professionalism made them appear distant: “But when they are too professional… He keeps a distance; he doesn’t ask many questions [or conversation]. You say to yourself, I will not come here again” (Ariadne).

If a Greek clinician was unavailable, most participants (*n* = 8) agreed that an interpreter would be beneficial, both in terms of communication and the comforting presence of another Greek. Two participants, however, reported a lack of trust in the fidelity of the interpreter: “I am worried that the interpreter might be confusing and not say what I say. I had this experience once” (Kristos). Three participants expressed mixed needs and expectations of the interpreter. “They brought me an interpreter… I looked at him reluctantly at first. I think that I told them ‘I understand what you are saying to me. I may not be able to answer properly, but I understand. So, I don’t need an interpreter’” (Alexandra).

Nuances within the Greek language were also reported, with half of the participants commenting on how the language has evolved since they moved to Australia. Many participants faced difficulties comprehending the formal Greek vocabulary used by interpreters. As such, some participants were hesitant to rely on interpretation by a formally educated person who may not use the same expressions and colloquialisms: “You should use simple words… People use formal high school Greek [that] we don’t understand. People that have gone to university use words [that] we don’t understand” (Iris). When relying on English, most participants (*n* = 8) reported a limited medical vocabulary “If they were in English, maybe I would be overstretched, because my English is not that fluent… I can be that talkative to you because I use my mother language. In English, I will be more restrained... a little more nervous. I would feel… as we say in English uncomfortable.” (Alexandra). Although most participants were in favor of an interpreter, some (*n* = 3) had varying needs and preferred a flexible approach to interpretation, based on language ability and task type: “For the written tasks, I don’t need an interpreter. But for the oral ones, you know, if I don’t know some words, it will be difficult to catch the meaning” (George).

### Experience of Neuropsychological Assessment

Six participants reported enjoying cognitive assessment due to the novelty and appreciated how mentally stimulating the tasks were: “It was like a game for me. I liked it” (Ariadne). However, throughout the assessment half of the participants recalled that they became discouraged, or felt ashamed due to their limited formal education and no prior experience with test-taking: “When I got something wrong… I felt like a fool… like not deserving to know all the things” (Helen).

Despite being assessed by a Greek-speaking neuropsychologist, most participants felt daunted by the formal assessment process. As a result, they felt unable to ask questions or voice concerns to the clinician: “I didn’t tell him [the neuropsychologist] that I felt worried... He didn’t have much time… I could not start a conversation about my personal issues. He would lose time. He had to see other people, too.” (Ariadne). Indeed, only one participant felt comfortable enough to ask for help when needed, expressing the desire to use the cognitive assessment as an opportunity to learn more skills.

Although the purpose of the assessment was explained in Greek prior to commencing and participants understood they were participating in research, most participants (*n* = 8) did not understand the purpose of the tasks or the broader purpose of cognitive assessment: “I wasn’t sure what you are going to do with the tests, because I haven’t done anything like this before… I think these tests make the brain work and keep you active so you don’t get dementia. They test how old people are.” (Mary). Participants reported being unsure of their performance and the expected responses: “I was not sure about some of the questions, but I tried to do my best…. some of the tests were confusing, but some were good.” (Kristos).

Those who expressed a lack of familiarity with the test content and style of questioning also reported more difficulties during the assessment. Five participants found tests of general knowledge most difficult, whereas four participants found drawing tests most difficult: “I finished the six years of primary school. But for the next 60 years I haven’t used a pencil. How will I write? I don’t remember.” (Alexandra). Other difficulties encountered were with timed tasks. Some participants expressed that being timed increased their anxiety and negatively affected performance: “It was hard for me to tell you words in Greek and I feel pressure when you are timing me, and I think this makes me forget some words.” (Kristos).

When questioned about the preference for Greek or non-Greek clinicians in future assessments, only one participant reported they would feel comfortable with a non-Greek clinician. Other participants expressed that being assessed by a member of the same cultural group reduced their anxiety, as the assessment itself was unfamiliar and anxiety provoking. The assessment experience was further improved by informal conversation and rapport building. Those who engaged in conversation with a clinician reported experiencing less anxiety: “What helped me? Having a chat was entertaining. The questions… all of it… I got calmer somehow.” (Zoe). Participants suggested potential adjustments to administrative procedures. Five participants suggested that assessments be conducted at the home of the participant, as hospitals tend to be anxiety provoking “In the hospital, you can’t stop thinking that you might be a patient… Not scared, but you meet a lot of unfamiliar people and our minds sometimes don’t work well. The hospital is an unknown environment. At home, you know, you are more encouraged… the context is more productive.” (George).

## Discussion

The present study aimed to understand how a sample of older Greek Australians experienced neuropsychological assessment and examine how cultural attitudes and contextual factors affect this experience. Participants reported that a range of sociocultural factors, including level and quality of education, differences in learning opportunities between sexes, a challenging settlement history post immigration, previous experiences of prejudice, and level of acculturation were likely to affect neuropsychological assessment. Although individuals of Greek descent technically fall into the broader European cultural category, following their arrival to Australia, many Greek immigrants experienced prejudice and social marginalization by the Anglo-Australian majority (Stevens, 1974). Past research has established that ethnocentric attitudes held by the White majority can result in the expression of prejudice against White minority groups, primarily due to viewing the cultural practices of dissimilar groups as inferior in comparison to one’s own (LeVine & Campbell, 1972). This example of discrimination highlights the experience of Greeks when immigrating to countries populated by Anglo majorities and is consistent with past research, documenting xenophobic and paternalistic attitudes experienced by Southern European groups (i.e., Greek, Italian, Portuguese, and Spanish) following immigration to Northern Europe (Schönwälder, 2004), North America (Rigby & Seguin, 2018), and Australia (Jupp, 2001). In light of these factors, the pre-and post-immigration challenges experienced by Greek Australians led to fortifying trust within their community due to a deeper understanding of culture, language, safety, and a shared lived experience. Overall, a combination of these factors appears to have shaped their cultural expectations and values, ultimately influencing their engagement with the general medical system and neuropsychological assessment.

In the context of the broader medical setting, responses relating to cultural identity highlighted the importance of establishing rapport, particularly when the assessing clinicians were not Greek speakers. Many participants viewed the prospect of being evaluated by a non-Greek clinician in a negative light. Specifically, issues relating to language barriers and a failure to understand and appreciate cultural norms were perceived as distant and dismissive, ultimately impacting the building of trust and rapport. Despite being in Australia for over 50 years, many Greek Australian older adults continue to identify primarily as Greek (Georgiades, 2014), a theme that was consistently reported by the present sample. As a result, most participants expressed a preference for Greek-speaking clinicians, favoring their own in group over perceived outgroup members. Such a preference presents a significant challenge for clinicians who are perceived as members of the “outgroup,” highlighting the importance of building rapport with clients prior to the assessment. Findings from other studies have noted that failure to build rapport, language barriers, and limited understanding of cultural customs can negatively affect the development of a therapeutic relationship, leading to poor treatment adherence, use of preventative and screening services, delaying access to care, and ultimately poorer health outcomes (Al Shamsi, Almutairi, Al Mashrafi, & Al Kalbani, 2020; Pandey et al., 2021). Notably, although rapport was established between Greek neuropsychologists and participants in the present study, which appeared to facilitate comfort, it did not necessarily result in participants understanding the purpose of undergoing neuropsychological assessment. Many participants were motivated to participate in research for philanthropic purposes, despite not understanding that neuropsychological assessment measures can assist with identifying brain disorders. Similar findings were noted by Plitas and colleagues (2009), who reported that their older Greek Australian participants were also motivated to participate for philanthropic purposes and were not able to grasp the concept that cognitive assessments are a valid means of identifying cognitive impairment. These findings suggest that in the context of clinical assessment, older Greek Australians may apply suboptimal effort due to a limited understanding of cognitive assessment and placing limited value on the overall process.

Consistent with previous research carried out in South Africa (Aghvinian et al., 2020), the impact of limited formal education and the subsequent testing experience was widely discussed by participants. Several participants reported experiencing significant difficulty with several tasks during the formal assessment process, including visuoconstructional drawing tasks. This finding is consistent with performances observed in other educationally disadvantaged older immigrant groups who displayed significant difficulty in drawing tasks and using pencils, resulting in outcomes that were indicative of cognitive impairment (Nielsen & Jørgensen, 2013; Staios et al., 2022). In contrast, one participant (George) credited his confidence and familiarity with testing to having completed secondary school. With the exception of this participant, epidemiological and sociocultural research indicates that the level of education observed within the remainder of the participants in the present study is broadly representative of this cohort (Dardiotis, Kosmidis, Yannakoulia, Hadjigeorgiou, & Scarmeas, 2014; Hurley et al., 2013; Plitas et al., 2009). Given that most neuropsychological tests have been designed to evaluate skills that are typically fostered and reinforced through formal schooling (Manly et al., 1999), the use of most tests placed individuals with limited education at a disadvantage. Consequently, traditional neuropsychological measures may overestimate the degree of any deficits and lead to misdiagnosis when applied to such groups. Prior to immigrating, the current cohort endured decades of sociopolitical instability, including exposure to World War II (1939–1945) and the Greek Civil War (1946–1949), which affected the quality of education attained (Mazower, 1994; Noutsos, 2003). Therefore, obtaining information relating to actual time spent in school and the quality of education attained may assist with accurately interpreting results derived from a clinical assessment, as years of education or grade levels alone may not necessarily be the best indication of educational attainment (Chin, Negash, Xie, Arnold, & Hamilton, 2012; Manly, Jacobs, Touradji, Small, & Stern, 2002). These findings stress the need to interpret test performance in low-educated and acculturated groups with caution, particularly in cases where access to demographically focused normative data and tests is not available.

Lack of previous experience with test-taking also influenced participants’ self-perception of the abilities tested, subsequently impacting their level of confidence. Although some participants found the assessment stimulating, many were daunted by the process and expressed shame at not being able to complete specific tasks. Consistent with previous research, many participants were sensitive to the fact that due to disrupted education, and exposure to war and poverty, they had limited opportunity to learn the specific skills tested during the assessment (Plitas et al. 2009). Additionally, both sexes viewed men as more likely to outperform women on assessment tasks because women generally attained lower levels of formal education prior to immigrating (Kosmidis, Tsapkini, Folia, Vlahou, & Kiosseoglou, 2004). However, results from our normative studies showed that men only outperformed women on three specific tasks, namely the Information, Digit Span, and Arithmetic WAIS-IV subtests (Staios, Kosmidis, Nielsen, et al., 2023). In contrast, women outperformed men on the supermarket verbal fluency test (Staios, Kosmidis, Kokkinis, et al., 2023). These pre-existing assumptions relating to sex difference and intelligence are likely due to several factors, including that most women were expected to care for their younger siblings and engage in other domestic duties in early childhood (Kosmidis et al., 2004) and most continued to engage in the traditional stay at home caregiving roles post immigration (i.e., raising children and managing the household), which limited their interaction with the broader society (Plitas et al. 2009). In contrast, most men had the opportunity to interact with peers and the workforce, which may have resulted in maintaining or enhancing skills attained from early schooling (Plitas et al. 2009). Therefore, sex disparities in socially reinforced skills and preexisting assumptions may be reflected in client confidence and test outcomes, potentially leading to overestimating cognitive impairment in older Greek Australian women.

In the absence of a Greek-speaking neuropsychologist, most participants responded favorably to the presence of an interpreter. However, some were hesitant and feared that they may be inaccurately represented. Furthermore, most participants commented on how the Greek language has evolved over the last several decades and that they may be at a disadvantage in instances where interpreters used formal Greek, recommending the use of simple language during the assessment to ensure clarity. Notably, there were differences in what participants thought constituted a need for interpretation. Some participants found the presence of an interpreter unnecessary given that a proportion understood what was being asked of them. Others, however, had difficulties expressing themselves and simply preferred additional time to formulate responses. Given the limited number of qualified multilingual neuropsychologists and logistic difficulties with referring clients onto a culturally similar neuropsychologist, interpreter-assisted assessments are often seen as a solution to overcoming language barriers. This topic has been a source of substantial debate and criticism, given the potential for introducing an additional source of error into an already complex situation (Fortuny et al., 2005; Fortuny & Mullaney, 1997, 1998). Irrespective of the methods used, Cagigas and Manly (2014) draw attention to the need for both cultural and clinical competency when assessing vulnerable groups in high-stakes situations, including considering client preference, comfort, and language proficiency when determining the need for an interpreter and level of involvement during the assessment.

**Fig. 2:**
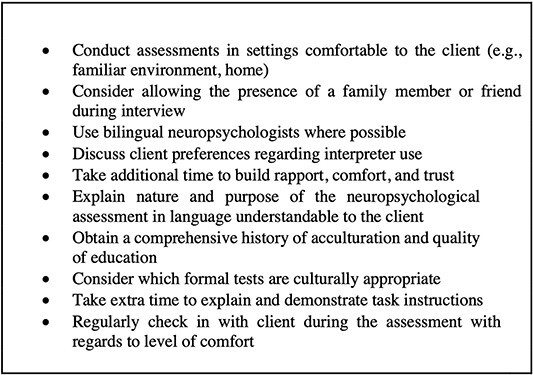
Key recommendations to create a culturally safe environment for neuropsychological assessment.

The findings arising from the present study have several implications for clinical practice. Additional care must be exercised when assessing educationally disadvantaged culturally diverse groups, particularly in the absence of appropriate tests and demographically focused normative data. Given that access to culturally appropriate tests and norms for individuals with low levels of education are generally limited, interpreting test outcomes must be done so with extreme caution. Furthermore, cultural competency and safety are key factors that warrant consideration within the context of a clinical assessment, particularly when assessing groups who have experienced prejudice and marginalization. The importance of creating a culturally safe environment is a key factor that may improve the delivery of clinical services. A failure to understand cultural nuances and to exercise cultural sensitivity when assessing culturally diverse groups is likely to have a negative impact and compromise the validity of neuropsychological assessment outcomes. As noted by the present sample, the need for perspective taking and cultural sensitivity appeared to be key factors in establishing rapport and alleviating anxiety throughout an unfamiliar situation, such as a cognitive assessment. In other words, initially “breaking the ice” by displaying appropriate self-disclosure and showing an interest in the client’s cultural background may assist with establishing rapport and improving the outcome of a cognitive assessment. Refer to Fig. 2 for key recommendations (Fujii, 2018).

The present study had several strengths worthy of mention. This is the first Australian study to comment on the role of cultural attitudes and contextual factors that may affect the validity of neuropsychological assessment outcomes, particularly in an older, culturally diverse group with limited education. Furthermore, we employed a qualitative framework that allowed for the extrapolation of first-hand nuanced themes relating to participant experiences that may serve as a foundation for future research examining cross-cultural neuropsychological phenomena not only in Australia but also internationally. However, several limitations within this study are also acknowledged. First, although we engaged in critical reflection in an attempt to mitigate cultural bias and preexisting beliefs, the unavoidable limitation of Greek-speaking clinicians assessing Greek-speaking participants may have had an impact on the interpretation of the data. Second, the time between the assessment and the interview differed across participants; therefore, disparities in the richness of recollections relating to the neuropsychology assessment may exist. It is possible that some recollections focused more broadly on participants’ general experiences with the medical system and immigration process, particularly for those interviewed where greater periods of time had lapsed since assessment. However, interviews began by reorientating participants to the neuropsychological assessment to assist with recalling specifics relating to testing. Third, participants were healthy and recruited from the general population for research purposes; therefore, experiences may differ from those experienced by people undergoing neuropsychological assessment for medical purposes. Assessments were conducted by Greek Australian neuropsychologists within participants’ homes for research purposes and may not reflect the further heightened levels of anxiety and experiences of those undertaking neuropsychological assessment with clinicians from different cultural groups in clinical settings. Finally, participants were interviewed in their own homes by a clinician who was also a member of their cultural group. We believe that a combination of these factors facilitated an optimal environment in which participants were able to openly discuss their subjective experiences. However, it is also possible they may have responded in a manner intended to be more socially desirable and minimized negative opinions or experiences. Indeed, participants’ reports of undergoing cognitive assessment may have differed if interviews were conducted by an English-speaking nonclinical researcher in a hospital setting. That being said, the latter scenario presents several barriers to engagement and depth of interview responses, particularly in instances where professionals are culturally and linguistically dissimilar (Graham, Halabi, & Nadler, 2021; Molina & Kasper, 2019).

In the context of cognitive assessment, research suggests that the effects of culture are likely to be most pronounced when cultural background is most divergent from Western culture (Ardila & Moreno, 2001). Although several differences exist, Greece and Australia share many sociocultural similarities rooted within a Western cultural mindset. However, despite this, most participants still reported the experience of undergoing cognitive assessment as foreign and anxiety provoking. As a result, one can only imagine how confronting the experience of undergoing a neuropsychological assessment might be for other individuals where there is an even greater cultural divide between the clinician and client. During the past decade, several developed nations have seen an influx of asylum seekers and refugees from areas of conflict, including Afghanistan, Syria, sub-Saharan African regions, and more recently Ukraine (United Nations Refugee Agency, 2022). Indeed, cultural diversity within nations, such as North America, Western Europe, and Australia has steadily increased over the past decades (United Nations, 2019). As these immigration data indicate, there is a growing need to make neuropsychological assessment more accessible and acceptable for culturally and linguistically diverse populations. However, research has observed that neuropsychological assessment of such groups can be extremely challenging, even when referring to existing best practice guidelines (Veliu & Leathem, 2016), highlighting ongoing barriers concerning accessing equitable services. Consequently, many immigrant groups are at risk of being inappropriately assessed, diagnosed, and treated (Nielsen & Staios, 2023).

In conclusion, this study explored the experience of Greek Australian older adults undergoing neuropsychological assessment and sought to understand the impact of cultural and contextual factors on cognitive assessment outcomes. The process of undergoing cognitive assessment was largely unfamiliar and anxiety provoking; however, factors such as rapport building, cultural discourse, and the presence of a bilingual speaker moderated these issues, to a certain extent. Although momentum is gaining, practical explicit guidelines on how to overcome the challenges of working with culturally and linguistically diverse populations remain limited (Fujii, 2023). Further research advances will be necessary to develop appropriate resources to assist clinicians with conducting culturally informed assessments to ensure fair and equitable outcomes.

## Data Availability

For legitimate research purposes data collected during this investigation will be made available by the authors, with personally identifiable information excised.

## Appendix

### Interview Schedule

1. Tell me what you did with the clinician that assessed you?
  - Why do you think you did these tests today?
  - What were your expectations beforehand?
2. How did you feel about today’s session? Probe: Motivated? Enjoyable? Stressed? Anxious? Confused? Unsure of yourself?
3. What did you like about doing these tests?
4. What did you not enjoy doing with these tests?
5. What kind of skills/things were these tests testing?
6. Did you understand the test instructions given to you?
  - What did you/did you not understand?
7. How do you think you did on these tests?
8. If you had to complete these tests in a hospital would you prefer to have an interpreter assist with test administration?
9. If you did not know the person (clinician) or if they could not speak Greek would that make a difference to your experience?
10. Is there anything that could have been done during the assessment that would have made you feel more comfortable?
11. Is there anything else that you would like to tell me about today?
